# Comparing Statistics and Machine Learning to Detect Insincere Grip Force Testing Using Manugraphy

**DOI:** 10.7759/cureus.33837

**Published:** 2023-01-16

**Authors:** Marion Mühldorfer-Fodor, Eren Cenik, Peter Hahn, Karl J Prommersberger

**Affiliations:** 1 Hand Surgery, RHÖN-KLINIKUM Campus Bad Neustadt, Bad Neustadt an der Saale, DEU; 2 Radiology, MVZ Rotenburg a.d. Fulda, Rotenburg an der Fulda, DEU; 3 Hand Surgery, Vulpius Klinik, Bad Rappenau, DEU; 4 Elective Hand Surgery, Krankenhaus St. Josef, Schweinfurt, DEU

**Keywords:** hand functions, value of statistics, manugraphy, grip force, machine learning, predictive model, malingering, load distribution, insincere effort, grip force testing

## Abstract

Background

Currently, there are no tests that have been proven to be capable of rating an individual’s grip force measurement as sincere or insincere. However, different parameters have been found to vary in grip force testing for maximal versus submaximal effort. A novel data analysis and processing approach might be key to improving these measurements. This study explores the use of a machine learning (ML) algorithm as a means to more accurately determine the sincerity or insincerity of grip force testing. The ML algorithm compares the hand’s load distribution pattern with the information generated using conventional statistical methods.

Methodology

This study uses manugraphy data collected as part of a previous investigation that analyzed load distribution patterns of the right and left hands of 54 healthy subjects. The subjects underwent grip force testing using maximal or submaximal effort, and the percentage contributions of each of the seven defined anatomical areas of the hand were calculated with respect to the total load applied. The predictions based on the load distribution and its use for rating individual grip force measurements as sincere or insincere were compared with the results of conventional statistical methods (thresholds for a bi-manual area-to-area comparison) and an ML algorithm.

Results

Based on an area-to-area comparison, our method achieved a sensitivity of 54% and a specificity of 78% to detect insincere effort. A predictive ML model developed using these data was capable of recognizing submaximal effort based on the hand’s load distribution pattern, determining a sensitivity of 94% and a specificity of 99%.

Conclusions

Compared to conventional methods, the use of an ML algorithm considerably improved the validity of manugraphy results in discerning the sincerity or insincerity of grip effort.

## Introduction

Grip strength is an important parameter used to evaluate general health status and, in particular, hand function. The results of grip strength tests are used in critical treatment decisions and are also essential criteria in assessing treatment results. Grip force measurements are commonly used to rate patient performance and adjudicate permanent disability with respect to work incapacitation, military duties, and/or claims for workmen’s compensation [1,2]. The accuracy of grip force testing depends directly on patient cooperation and their willingness to exert maximum effort. Patients who claim losses or reductions in grip force in the absence of objective findings accounting for such disabilities are frequently evaluated with extensive diagnostic workups; they may also receive ineffective and unnecessary therapeutic procedures that prolong their absence from the workplace [2-5]. However, there are no reliable methods that can be used to detect insincere effort; thus, this problem remains largely unsolved. Improved tools and methods designed to evaluate the degree of effort and sincerity in grip strength testing are much needed by practitioners in this field.

The existing methods such as the JAMAR dynamometer, the five-rung test [4,6], the rapid exchange grip test (REG) [7,8], and the coefficient of variation [3,9] provide only minimal hints regarding submaximal effort used in grip force testing. Electronic devices facilitate the generation and analysis of a force-over-time curve associated with grip force testing based on various parameters, for example, the time required for grip initiation, the force-generation phase, the force-decay phase, or the time required for grip release [10,11]. However, these tests are not sufficiently sensitive or specific in determining whether an individual test result is most likely sincere or specifically insincere [10,12-18].

While each of the aforementioned methods has been used to generate statistically significant differences between groups of individuals tasked with performing maximal or submaximal grip force testing, there are problems associated with attempting to rate an individual test result as sincere or insincere. Some studies showed promising results when evaluating healthy and strong subjects with these methods but failed with truly weak patients [19,20]. Efforts to define universal threshold levels for patients with various pathologies were complicated by individual grip force levels that varied widely and intra-individual physiological performance fluctuations. Therefore, we hypothesized that the assessment methods themselves might not be the problem but that conventional calculations based on defined thresholds might not be effective for analyzing complex data and might impede the reliable clinical application of these methods.

Results from a previous study revealed that the load distribution pattern of the hand, as assessed by the Manugraphy system®, changes when comparing grip force exerted with sincere versus insincere submaximal effort [21]. The Manugraphy system® (Novel GmbH, Munich, Germany) includes a set of cylinders covered by a soft matrix with embedded pressure sensors that can measure the total grip force. Graphs are used to present a matrix with all sensors and their pressure values in the form of a “load-distribution map” that describes the contact area between the hand and the cylinder surface. The Manugraphy system® provides a quantitative assessment of the load distribution at each contact point. The contact area was divided into seven representative areas: the thumb, each of the four fingers, and the thenar and hypothenar eminences. The percent contribution from each area was then calculated. Previously published manugraphy studies revealed that while the load distribution varied among individuals, it was similar when comparing both hands from a single individual [22,23]. By contrast, a more recent study documented highly significant intra-individual differences between the left and right hands of a single individual when each is responding with maximal and submaximal effort [21].

Until now, load distribution data were analyzed through an area-to-area comparison. In other words, the loads applied by each of the seven areas of one hand while performing a grip test with maximal effort were compared to the corresponding areas of the opposite hand that were applying only submaximal effort. This study revealed that five and seven of the total seven corresponding areas differed when comparing the results of the left and right hands that engaged in maximal and submaximal efforts, respectively [21]. The data collected from all participants were integrated and used to perform a statistically valid between-group comparison. For clinical applications, however, there needs to be some way to determine whether the results of an individual test are reliably categorized as sincere (maximal) or insincere (submaximal). To achieve this, conventional methods typically refer to threshold limits, which may or may not be accurate in this case due to widely differing individual grip force levels and intra-individual physiological performance fluctuations, such as those documented using the JAMAR dynamometer.

The simultaneous evaluation of all seven areas of the hand results in a specific manugraphy pattern. Efforts to detect similarities and periodic combinations that contribute to patterns based on multiple variables require the analysis of an enormous amount of data. This information may be processed very effectively by machine learning (ML), which is a field of computer science that evolved from artificial intelligence and pattern recognition. ML computer programs are designed to “learn” patterns without being explicitly programmed; thus, ML algorithms may ultimately provide insights into the nature of individual grip patterns regardless of the variations in individual characteristics. Essentially, ML can complete different tasks related to regression and classification. Regression is used to predict continuous outcomes, such as temperature and prices, while classification predicts non-continuous outcomes or classes, such as high vs. low and red vs. blue vs. green.

Accordingly, this study aimed to determine whether ML is more accurate than conventional threshold-based methods in identifying submaximal grip force (sincere or insincere) through an analysis of load distribution patterns.

## Materials and methods

The Manugraphy system®

**Figure 1:**
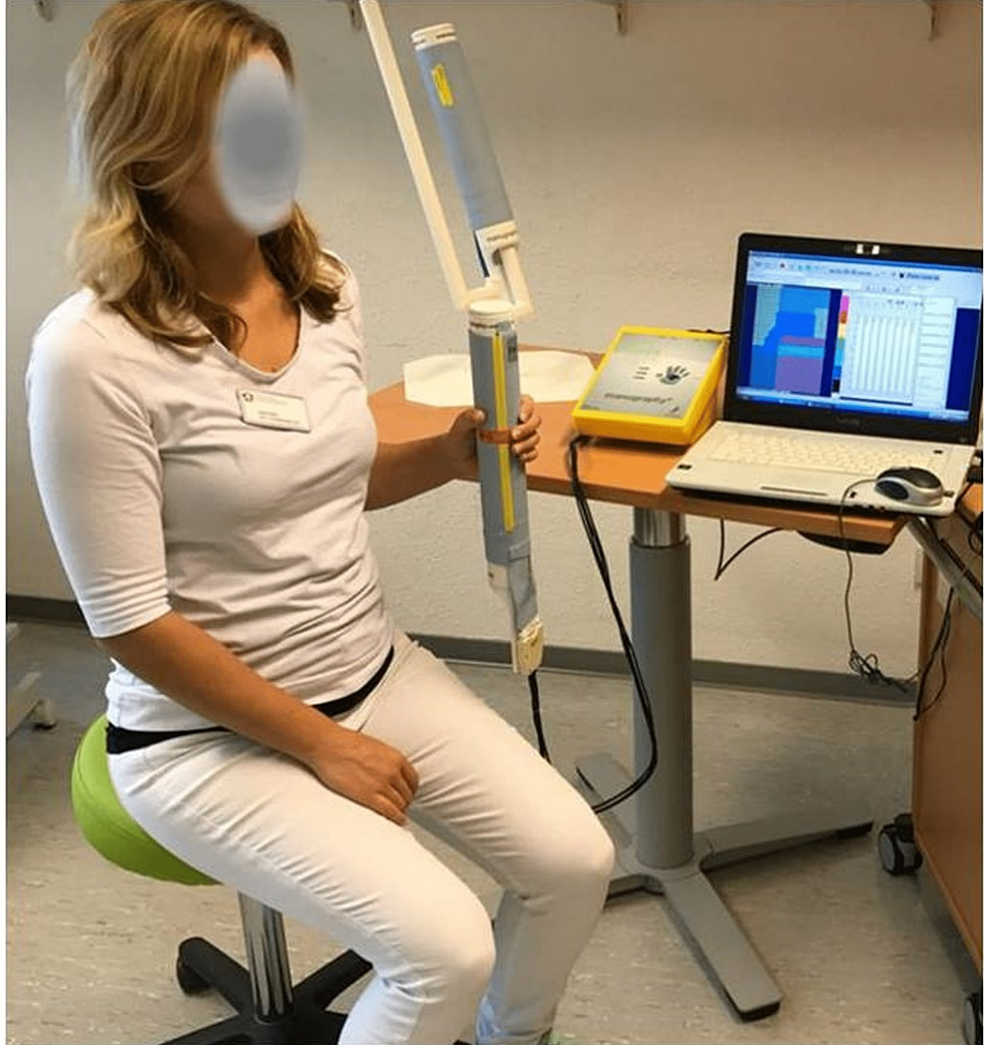
The Manugraphy system® used to measure grip force and analyze the load distribution of the hand. Tests need to be performed in a standard position recommended by the American Association of Hand Therapists. Variants in posture, such as a different forearm rotation, might influence the load distribution of the hand. Image credit: Marion Mühldorfer-Fodor

**Figure 2:**
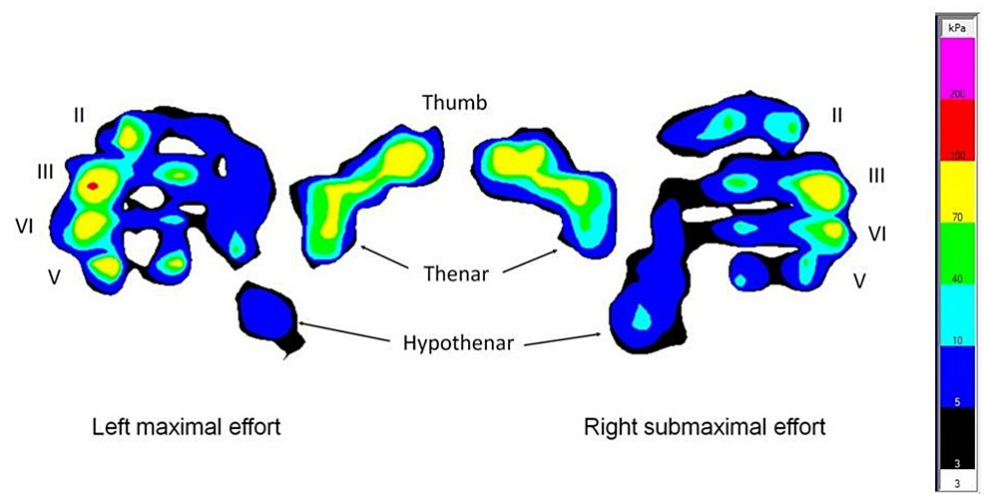
When gripping a Manugraphy® cylinder, the points of contact with the hand are visualized by the load applied to the pressure sensors. Specific colors are used to represent the load values (see the scale on the right side). This illustration documents the load distribution of both hands with the right hand exerting submaximal effort. With maximal effort, the fingertips of the index (II), ring (IV), and small finger (V) apply similar loads, with the tip of the middle finger (III) at the peaks. In the cases of submaximal effort on the right side, the index and small finger are neglected, while the middle and ring finger apply similar loads. While the load exerted by each of the fingers decreases with submaximal effort, increases are detected at the hypothenar eminence. Image credit: Marion Mühldorfer-Fodor

Additional technical information on this system can be found in previous reports [22,24]. Briefly, a soft sensor matrix covers the Manugraphy® cylinder (Figure 1), continuously covered with pressure sensors with a size of 7mm2, providing a spatial resolution of two sensors per cm². The software calculates the force component perpendicular to the cylinder surface for each sensor; this is displayed as a raster diagram with a color scale and numerical values (Figure 2). To provide a quantitative analysis of the load distribution, the contact area was divided into seven representative areas based on anatomical landmarks (i.e., the thumb, the four fingers, the thenar, and the hypothenar). The percent contribution of each area to an individual’s total grip force can then be calculated.

Datasets

This cross-sectional study uses manugraphy data published in a previous study [21]. The basic investigational parameters are as follows: 54 healthy subjects (25 males and 29 females; 52 right-handed and two left-handed) with a mean age of 34 years (range: 19-53 years) participated in this study. The study group was recruited from the members of the hospital staff, including surgeons, physical and occupational therapists, secretaries, and students. The subjects performed grip force tests on two separate visits using the 200 mm cylinder of the Manugraphy system® to measure grip force and load distribution. The participants were instructed to grip the Manugraphy® cylinder over three intervals of five seconds each (trials 1-3); each trial was followed by a 10-second break. The subjects were asked to grip the device with maximal force with one hand and submaximal force (i.e., ½ to ¾ strength) with the opposite hand to mimic the conditions relevant to feigned weakness. The hand used to exert submaximal effort during the first session was determined by an online randomizer; the opposite hand was examined during the second session.

**Figure 3:**
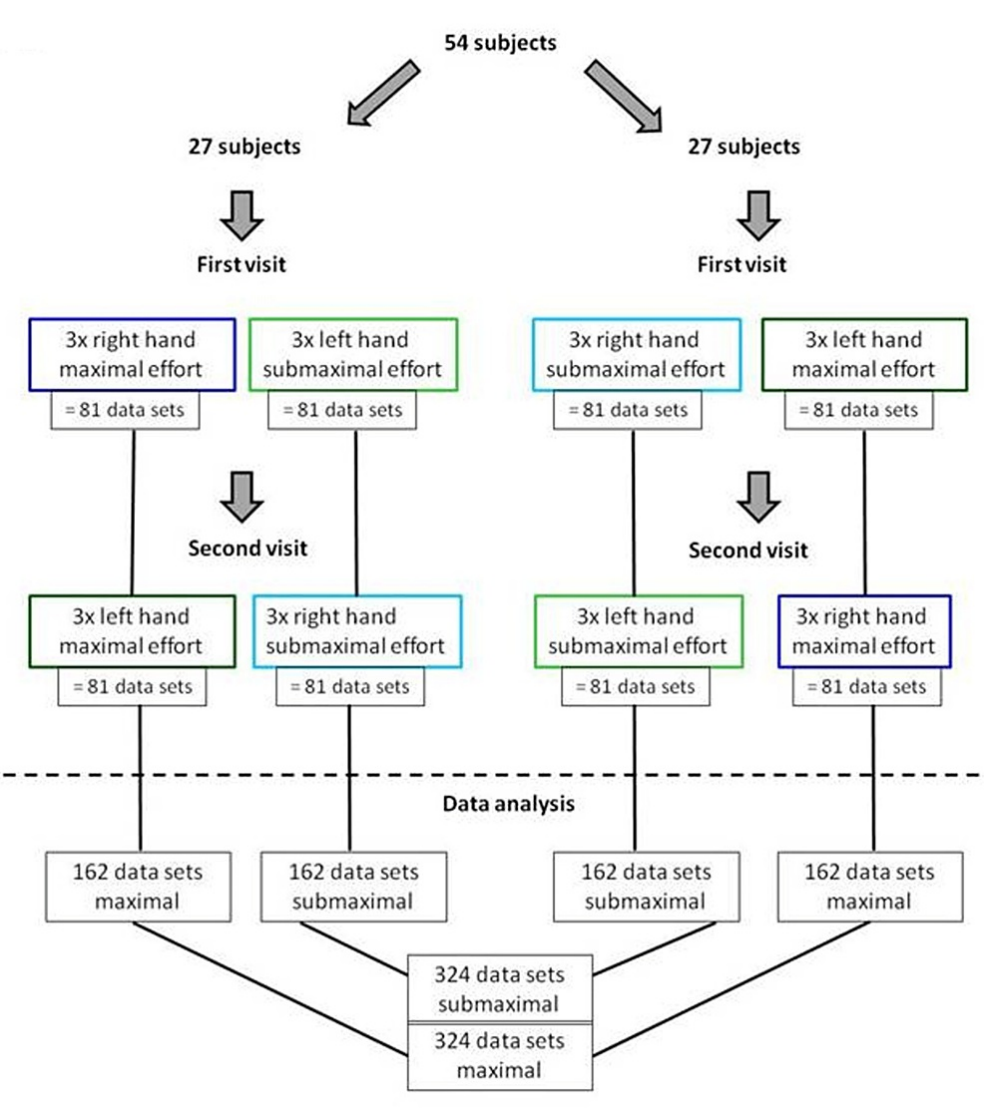
The flowchart illustrates the acquisition of datasets for maximal and submaximal efforts. Image credit: Marion Mühldorfer-Fodor

**Figure 4:**
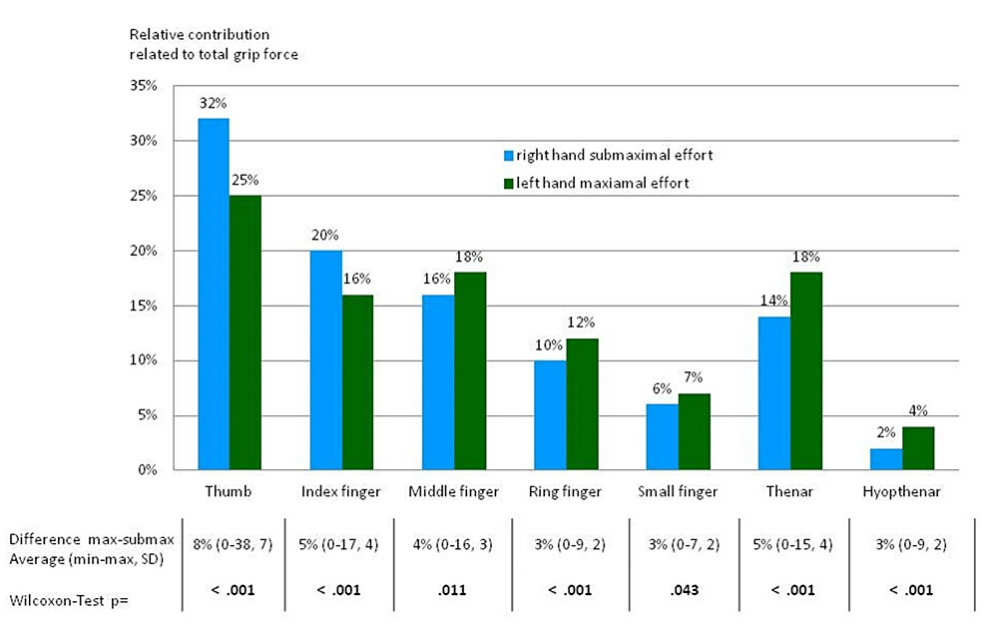
The figure presents the percent contribution of each area of the hands to the total load assessed in 54 subjects; For the calculation shown, the left hand exerted maximum effort, while the right hand exerted submaximal effort. The corresponding areas were compared to one another using the Wilcoxon test. Average differences between the right and left hands are shown with minimum and maximum values and standard deviations. On average, the thumb and the index finger were neglected the least and transmitted more force in relation to the rest of the hand when an insincere effort was performed. The thenar was mostly neglected. Image credit: Marion Mühldorfer-Fodor

**Figure 5:**
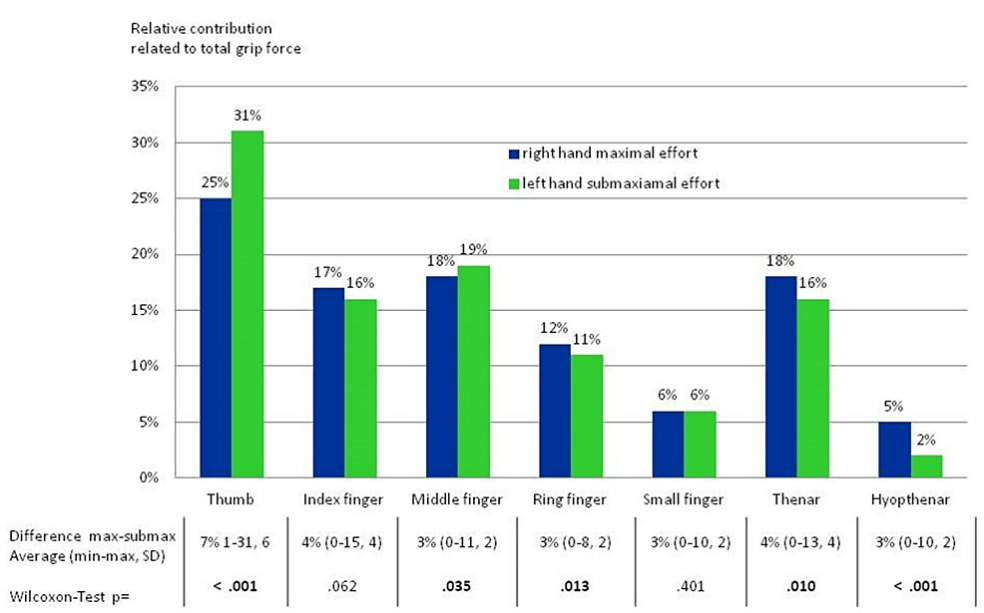
This figure shows data when the right hand performed grip at maximum capacity, while the left hand exerted submaximal effort. On average, the thumb was neglected the least and the hypothenar was neglected the most. Image credit: Marion Mühldorfer-Fodor

The analysis and basic statistics were used to evaluate load distribution. As noted above, the study protocol provided data from 54 subjects who performed a full set of maximal and submaximal grip force tests using both hands. Each subject performed three trials per visit. Thus, the final results included 324 datasets with maximal grip effort and 324 datasets with submaximal effort (Figure 3). The percent load-contribution values of the seven anatomical areas and the total grip force were calculated for further analysis. Results from each of the seven areas on one hand (i.e., maximal effort) were compared to the results from the same areas on the opposite hand (submaximal effort) using a Wilcoxon test. This area-to-area comparison documented statistically different load-distribution patterns when comparing the results of submaximal with those of maximal effort (Figure 4 and Figure 5) [21].

Categorization of (blinded) individual results

**Figure 6:**
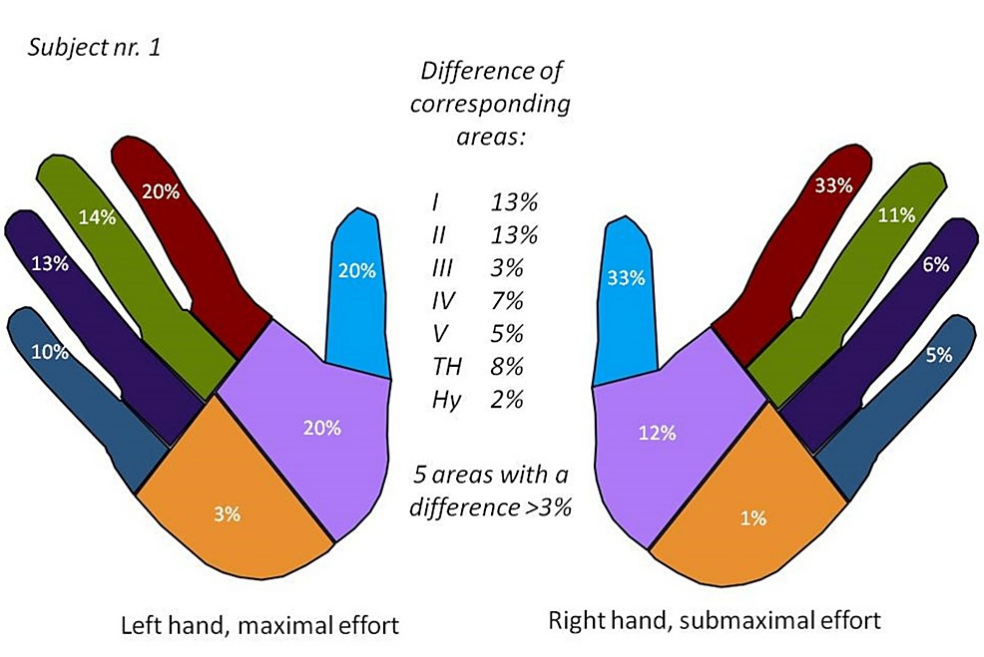
Load distribution resulting from a trial of exhibiting maximal effort with the left hand and submaximal effort with the right hand. In an area-to-area comparison, five (I, II, IV, V, and TH) of seven corresponding areas exhibited >3% differences. Symbols: I, thumb; II, index finger; III, middle finger; IV, ring finger; V, little finger; TH, thenar eminence; Hy, hypothenar eminence. Image credit: Marion Mühldorfer-Fodor

*For a conventional prediction model*, thresholds (i.e., cut-off values) had to be defined in order to categorize blinded load distribution data pertaining to the maximal or submaximal effort. By comparing patterns for one hand exerting maximal effort to those of the opposite hand exerting submaximal effort, the differences in percent contribution of the corresponding areas could be calculated for each subject. Previous manugraphy studies revealed that this testing may be complicated by some physiological performance differences between the hands; differences of >3% were considered to be significant [22,24]. Thus, data from each subject were analyzed based on the number of anatomical areas exhibiting a difference of >3% when comparing both hands (Figure 6). Once this was established, the sensitivity and specificity used to categorize the blinded load distribution values correctly as sincere or insincere were calculated for different cut-off values corresponding to areas differing by >3%, as described above.

To create a* prediction model *using ML, we converted grip force and load-contribution values to logarithms because of the skewness of the data. Initially, it was unclear which information might be useful for ML-based calculations and algorithm creation; thus, several features and combinations of features were tested. Besides the load contribution of the seven areas described above, their mean, variance, and standard deviation, both with and without logarithmic conversion, were selected as critical features.

The next step was model training, where 80% of the data were used to create a model and the remaining 20% was used to validate the model’s accuracy. Both datasets were chosen randomly by the software with an equal distribution of maximal and submaximal observations. Standard five-fold cross-validation was performed during model training to avoid overfitting, and parameter tuning was performed by a grid search.

The data were analyzed using various ML algorithms, including Random Forest, Support Vector Machines, Gradient Boosting Algorithm, and XGBoost [25]. The final model was tested using the aforementioned validation dataset. The performance of the algorithm was calculated using the confusion matrix for predicted vs. true values. Specificity and accuracy were calculated from the confusion matrix [26]. Additionally, the receiver operating characteristic (ROC) curve was plotted, and the area under the curve (AUC) was calculated.

## Results

Conventional data analysis

**Figure 7:**
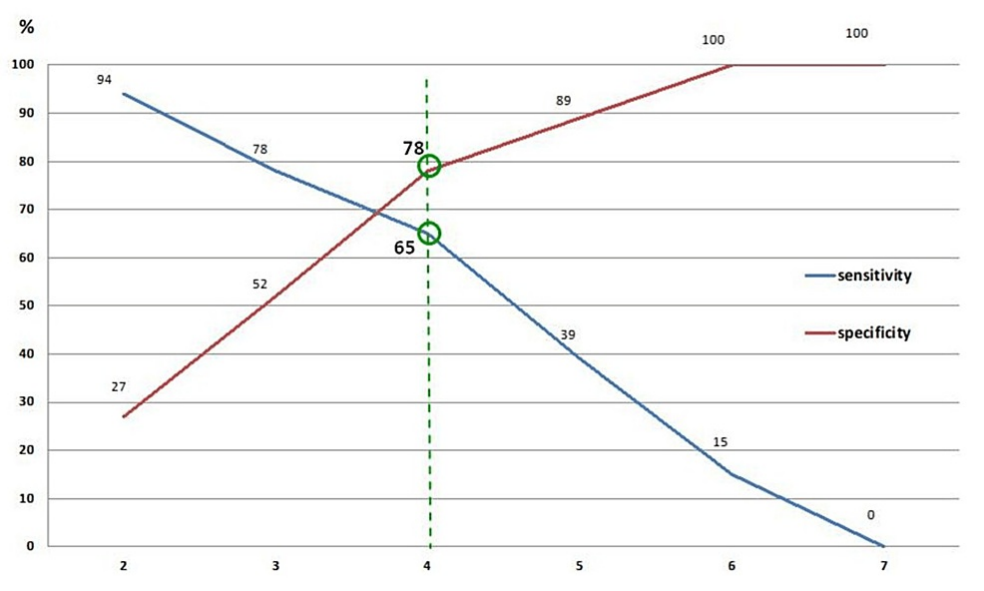
A graph depicting corresponding sensitivity and specificity based on varying threshold limits (i.e., the number of corresponding areas that differ by >3%). Image credit: Marion Mühldorfer-Fodor

The ROC curve was calculated based on varying numbers of corresponding areas that differed by >3% to label the blinded load distribution results as “insincere” cut-off values. The most appropriate cut-off value was identified for four areas that resulted in a sensitivity of 54% and a specificity of 78% (Figure 7).

Machine learning

The following 13 criteria were identified as relevant to this process: individual age, sex, right-/left-handedness, total grip force, percent load on the index finger, middle finger, ring finger, small finger, hypothenar, and thenar, the statistical variance of the load on the seven identified anatomical areas, and the logarithm of their sum [27]. All data analyses were performed in R (R Core Team 2013).

From the algorithms tested, the XGBoost ML learning algorithm generated the best results (Table 1). A submaximal effort could be predicted with a sensitivity of 0.938, a specificity of 0.986, and an accuracy of 0.98 (95% CI, 0.9635-0.9885). The evaluation of the ROC curve resulted in an AUC of 0.995.

**Table 1:** Results of conventional data analysis and ML algorithms. AUC = area under the curve; CI: confidence interval

Method	XGBoost	svmlinear	gbm	Random Forest	Thresholds
					4 areas differing
Sensitivity	0.9381	0.8351	0.8557	0.8660	0.5370
Specificity	0.9862	0.9783	0.9783	0.9783	0.7778
Accuracy	0.9785	0.9553	0.9586	0.9603	0.6173
(95% CI)	(0.9635, 0.9885)	(0.9356, 0.9703)	(0.9395, 0.973)	(0.9415, 0.9744)	(0.5378, 0.6924)
AUC	0.995	0.982	0.9907	0.9934	0.6574
(95% CI deLong)	(0.9914-0.9987)	(0.9707-0.9934)	(0.855-0.996)	(0.9891-0.9977)	0.5842-0.7306

## Discussion

This study investigated the potential of ML in categorizing grip force testing with maximal or submaximal effort using load distribution data generated by the Manugraphy system®. Conventional methods using thresholds for bimanual area-to-area comparisons were compared to the execution of algorithms created by ML methods with respect to their capacity to identify insincerity (i.e., the use of submaximal effort) during grip force testing. Conventional methods failed to predict insincere effort with reliable results (sensitivity of 54% and specificity of 78%). By contrast, this study revealed the power of ML algorithms used to make accurate predictions and differentiate between the insincerity and sincerity of the efforts of each individual undergoing grip force testing. The ML algorithm detected 94% of all tests performed with submaximal effort and misclassified only 1% of the maximal strength trials. An additional advantage of the ML algorithm is that the load distribution of one hand can be evaluated without the need to compare it to the opposite hand.

Currently, there are no reliable and commonly accepted tests that might be used to differentiate between sincere and insincere efforts in grip force testing; thus, it is not possible to determine whether a patient is truly weak or is feigning grip force loss [28,29]. Some fundamental problems inherent to all previously presented methods on this topic include the need to assess individual responses rather than group outcomes, Numerous studies have indicated changes and even statistically significant differences between groups when comparing maximal effort and feigned grip force loss during grip force testing. However, using this information to determine whether an individual’s test score was the result of sincere or insincere effort has consistently failed. This problem may be associated at least partly with variability in individual grip characteristics and the normal physiological fluctuations in grip force. For all tests, the investigators chose threshold levels for specific criteria (i.e., cut-off values) used to rate a test result as insincere. Notably, less stringent criteria yielded lower sensitivity and greater specificity, while more stringent criteria yielded the reverse properties [13]. A ROC curve depicts the link between different sensitivities and specificities observed by using different cut-off values. The most appropriate pairing and corresponding threshold levels can be determined based on the test aim. With respect to efforts to identify insincere grip force loss, mistaken labeling of feigning patients as sincere may have considerable clinical and financial consequences, including the performance of unnecessary diagnostic and therapeutic procedures, as well as increased disability and healthcare costs. However, labeling sincere individuals as insincere can result in inappropriate diagnosis and inadequate treatment for the patient concerned, which may result in withheld payments, reduced worker’s compensation, and even job loss. As physicians, our code of medical ethics implies a responsibility toward every patient. Considering this, we concluded that it is better to err in the direction of low sensitivity because unfairly misclassifying a sincere subject as insincere and promoting clinically unfair decisions can be detrimental to the individual in concern [13]. None of the previously published methods could provide a predictive value appropriate for clinical use based on conventional calculations. Thus, the results of this study confirm our hypothesis that the problem may not be the assessment method itself but the fact that conventional calculations based on thresholds might not be appropriate for the analyses of complex data. Thus, the use of the hand-based load distribution calculator to detect insincerity during grip force testing, followed by data analysis and interpretation using a computed ML algorithm, seems to be an effective method. This calculator may also be used to analyze data from the five-rung test, the rapid exchange grip test, or force-over-time curves using a digital JAMAR dynamometer. However, this study also demonstrated the possibilities offered by ML to improve the analysis of clinical data and provide a more accurate prediction than conventional statistics.

This study represents the first attempt to analyze manugraphy data using an ML algorithm [27]. The sensitivity and specificity resulting from the use of this method were superior to those achieved using conventional methods. The presented ML algorithm is only the first step toward the development of a trustworthy and comprehensive test procedure. ML programs are capable of creating even better algorithms once they have been provided with additional data that can be used for model training, which allows for even greater accuracy. Further research is needed to explore the limitations of this approach. Moreover, this study only included healthy individuals. More research is needed to train the ML model with data on grip testing of patients using different hand, wrist, and/or upper extremity pathologies in order to evaluate the model’s predictive value for clinical application.

The method developed in this study has some limitations. The evaluation depends completely on the computer algorithm used to perform calculations that remain invisible and not fully comprehensible to the user or subject undertaking the test. We recognize that while some changes in the load distribution pattern are obvious on visual inspection (Figure 2), the definitive rating generated by the algorithm remains a virtual “black box” in the testing situation [30].

Further, this method is not applicable when pathologies modify the load distribution of the hand, such as motor palsy, tendon pathologies, and scar restriction. However, grip force loss in these cases is usually not contested. This method is appropriate for persons who claim losses or reductions in grip force in the absence of objective findings indicating such disabilities, for example, minor trauma and correctly healed fractures. 

Another drawback is that this method is highly dependent on the availability of specific technical resources. The Manugraphy system® is commercially available but is not in wide use yet. Furthermore, the algorithm developed is also not widely available yet. Currently, direct data transfer from the Manugraphy system® to the ML program is cumbersome and time-consuming. As the next step, the algorithm needs to be integrated into the measurement tool if there is any possibility of its clinical application. As a more distant goal, greater sensitivity and specificity might be achieved by eliminating the seven hand sections contributing to the distribution pattern and instead analyzing the distribution pattern sensor by sensor. Depending on the size of the hand and the cylinder, 120-200 sensors are typically activated while gripping a cylinder.

## Conclusions

Compared to conventional methods, the use of ML led to considerable improvements in the predictive value of manugraphy results used to rate the sincerity of an individual’s grip effort. This study emphasizes the limitations of conventional statistical methods in rating individual test results even when the test parameter differed significantly in group-to-group comparisons. Likewise, conventional calculations using thresholds might not be appropriate for parameters that vary substantially among individuals and are subject to physiological fluctuations during test repetitions not only for grip force measurements but also for the evaluation of other clinical questions. ML might be a helpful approach to overcome these problems.
